# The Absence of PTEN in Breast Cancer Is a Driver of MLN4924 Resistance

**DOI:** 10.3389/fcell.2021.667435

**Published:** 2021-04-30

**Authors:** Meng-ge Du, Zhi-qiang Peng, Wen-bin Gai, Fan Liu, Wei Liu, Yu-jiao Chen, Hong-chang Li, Xin Zhang, Cui Hua Liu, Ling-qiang Zhang, Hong Jiang, Ping Xie

**Affiliations:** ^1^The Municipal Key Laboratory for Liver Protection and Regulation of Regeneration, Department of Cell Biology, Capital Medical University, Beijing, China; ^2^State Key Laboratory of Proteomics Beijing Proteome Research Center, National Center for Protein Sciences (Beijing), Beijing Institute of Lifeomics, Beijing, China; ^3^School of Medicine, Tsinghua University, Beijing, China; ^4^Shandong Provincial Key Laboratory of Pathogenesis and Prevention of Neurological Disorders and State Key Disciplines: Physiology, Department of Physiology, School of Basic Medicine, Medical College, Qingdao University, Qingdao, China; ^5^CAS Key Laboratory of Pathogenic Microbiology and Immunology, Institute of Microbiology (Chinese Academy of Sciences), Savaid Medical School, University of Chinese Academy of Sciences, Beijing, China

**Keywords:** MLN4924, UBA3, neddylation, PTEN, breast cancer

## Abstract

**Background:** Numerous studies have indicated that the neddylation pathway is closely associated with tumor development. MLN4924 (Pevonedistat), an inhibitor of the NEDD8-activating E1 enzyme, is considered a promising chemotherapeutic agent. Recently, we demonstrated that neddylation of the tumor suppressor PTEN occurs under high glucose conditions and promotes breast cancer development. It has been shown, however, that PTEN protein levels are reduced by 30–40% in breast cancer. Whether this PTEN deficiency affects the anti-tumor function of MLN4924 is unknown.

**Methods:** In the present study, cell counting kit-8 and colony formation assays were used to detect cell proliferation, and a transwell system was used to quantify cell migration. A tumor growth assay was performed in BALB/c nude mice. The subcellular location of PTEN was detected by fluorescence microscopy. The CpG island of the UBA3 gene was predicted by the Database of CpG Islands and UCSC database. Western blotting and qRT-PCR were used to measure the expression of indicated proteins. The Human Protein Atlas database, the Cancer Genome Atlas and Gene Expression Omnibus datasets were used to validate the expression levels of UBA3 in breast cancer.

**Results:** Our data show that the anti-tumor efficacy of MLN4924 in breast cancer cells was markedly reduced with the deletion of PTEN. PI3K/Akt signaling pathway activity correlated positively with UBA3 expression. Pathway activity correlated negatively with NEDP1 expression in PTEN-positive breast cancer patients, but not in PTEN-negative patients. We also demonstrate that high glucose conditions upregulate UBA3 mRNA by inhibiting UBA3 promoter methylation, and this upregulation results in the overactivation of PTEN neddylation in breast cancer cells.

**Conclusion:** These data suggest a mechanism by which high glucose activates neddylation. PTEN is critical, if not indispensable, for MLN4924 suppression of tumor growth; PTEN status thus may help to identify MLN4924-responsive breast cancer patients.

## Introduction

Breast cancer has overtaken lung cancer as the world’s most common cancer. Effective therapy of breast cancer requires precise treatments that are tailored to genomic status. Therefore, it is important to identify new diagnostic methods, drug targets and prognostic tools from the results of studies of the pathogenesis and molecular mechanisms underlying breast cancer (Waks and Winer, 2019; Hanker et al., 2020).

Phosphatase and tension homolog on chromosome 10 (PTEN) is one of the most frequently mutated genes in human cancers and inherited syndromes (Song et al., 2012). Absence of PTEN results in the activation of the phosphatidyl inositide 3-kinase (PI3K)/Akt oncogenic pathway, which controls cell growth and survival (Di Cristofano et al., 1998; Stambolic et al., 1998). Recently, we reported that PTEN is a novel target for modification with NEDD8. High concentrations of glucose trigger PTEN neddylation, resulting in PTEN nuclear import. In breast cancer patients, neddylated PTEN correlates with tumor stages and with a poor prognosis (Xie et al., 2021). NEDD8 is a ubiquitin-like protein (UBL) that is covalently conjugated to substrates in a manner similar to the ubiquitin system. The neddylation system includes an activating enzyme (E1, which consists of a heterodimer of UBA3 and NAE1/APP-BP1), two conjugating enzymes (E2s, which are known as UBE2M/Ubc12 and UBE2F), and various E3 ligases (Enchev et al., 2015). Neddylation is reversible through the deneddylases NEDP1 and JAB1/CSN5 (Cope et al., 2002; Mendoza et al., 2003).

An inhibitor of the NEDD8-activating enzyme E1, MLN4924 (Pevonedistat), has shown promise as an anti-cancer agent. Previous studies indicated that MLN4924 inhibits breast cancer cell growth and migration (Chen et al., 2018, 2020; Naik et al., 2020), and it displays potent preclinical activity for patients with acute myelocytic leukemia, acute lymphocytic leukemia, glioblastomas, Wilms tumors, rhabdomyosarcomas, and neuroblastomas (Soucy et al., 2009; Wang et al., 2011; Nawrocki et al., 2012). Accordingly, MLN4924 has been evaluated in a series of phase 1, 2, and 3 clinical trials, both alone and in combination with other chemo- and radiotherapies (Soucy et al., 2009; Abidi and Xirodimas, 2015). MLN4924 serves as a chemo- or radiosensitizer in pancreatic (Wei et al., 2012), colorectal (Wan et al., 2016), prostate (Wang et al., 2016), and ovarian (Nawrocki et al., 2013) cancer cells, and it has been found to be more effective in combination with other chemo- or radiotherapies, including azacytidine (Swords et al., 2018) and 2-deoxy-D-glucose (2-DG) (Oladghaffari et al., 2017). Unfortunately, loss or reduction of PTEN protein is common in numerous tumors, including breast cancer (Perren et al., 1999; Costa et al., 2020), and PTEN is thought to be a necessary factor for MLN4924 sensitivity.

Here, we specifically studied the role of PTEN presence in the biological activities of MLN4924. Our data showed that MLN4924 suppressed Akt signaling in a PTEN-dependent manner. Loss of PTEN particularly weakened the anti-tumor ability of MLN4924 in breast cancer. Furthermore, we found that high glucose inhibits UBA3 promoter methylation and increases UBA3 mRNA levels, and these outcomes correlate with the overactivation of PTEN neddylation in breast cancer cells. The PI3K/Akt signaling pathway is positively correlated with the expression of UBA3, but the correlation is not significant in PTEN-null breast cancer patients. Therefore, our data suggest that those patients with cancer that harbor complete *PTEN*-loss may be resistant to MLN4924. In addition, we suggest that low levels of UBA3 promoter methylation in breast cancer patients could suggest promising tumor therapeutic targets.

## Materials and Methods

### Cell Culture and Transfections

MCF-7, BT-549, MDA-MB-231, SKBR3, and T-47D were obtained from the American Type Culture Collection (ATCC). MCF-7 cells were cultured in DMEM (GIBCO-Invitrogen) supplemented with 10% fetal bovine serum (FBS). BT-549 and T-47Dcells were cultured in RPMI-1640 (GIBCO-Invitrogen) supplemented with 10% FBS. MDA-MB-231 cells were cultured in Leibovitz’s L-15 Medium supplemented with 10% FBS. SKBR3 cells were cultured in Dulbecco’s modified Eagle’s medium and GlutaMAX-1 (Gibco Life Technologies) containing 10% FBS. Cells were transfected with various plasmids using TuboFect (Thermo Fisher Scientific, R0531), Lipofectamine 3000 (Invitrogen, L3000001) reagent according to the manufacturer’s protocol.

### Antibodies and Regents

Antibodies used in this work: Anti-Akt (CST, #9272, 1:1,000), anti-pThr308-Akt (CST, #9275, 1:1,000), anti-pSer473-Akt (CST, # 4060, D9E, 1:1,000), anti-p70 S6K (CST, #9202, 1:1,000), anti-pThr389-p70 S6K (CST, no. 9209, 1:1,000), anti-pSer235/236-S6 (CST, #4858, 1:1,000), anti-S6 (CST, #2317, 1:1,000), anti-4E-BP1 (CST, #9452, 1:1,000), anti-pSer65-4E-BP1 (CST, #9451, 1:1,000), anti-PTEN (CST, #9559, 1:1,000), anti-pSer2448-mTOR (CST, # 5536, 1:500), anti-mTOR (CST, #2972, 1:500), anti-UBE1a (CST, # 4890, 1:1,000) and anti-pAMPK (CST, #2535, 1:1,000) were purchased from Cell Signaling Technology. Anti-GAPDH (Santa Cruz, sc-293335, 1:1,000), Anti-UBA3 (Santa Cruz, sc-377212, 1:200), anti-NAE1 (Santa Cruz, sc-390002, 1:200) and anti-NEDP1 (Santa Cruz, sc-271498, 1:100) were from Santa Cruz Biotechnologies. Anti-UBE2D3 (Abcam, ab176568, 1:1,000), anti-Cullin1 (Abcam, ab75817, 1:1,000) and anti-SAE1 (Abcam, ab185552, 1:1,000) were purchased from Abcam. Anti-Nedd8-K402-PTEN antibody was from PTM Biolabs, Inc. The NAE inhibitor MLN4924 (HY-70062), 2-Deoxy-D-glucose (a glucose analog and a competitive inhibitor of glucose metabolism) (2-DG, HY-13966), 5-Azacytidine (Azacitidine; 5-AzaC; Ladakamycin) (HY-10586) were purchased from MCE.

### RNA-Seq and Data Analysis

Total RNA was isolated using Trizol (Sigma, Saint Louis, MO) and cDNAs were synthesized by reverse transcription kit (Bio-Rad, Hercules, CA). The cDNA library products were sequenced on an Illumina HiSeq 2000 (Illumina, San Diego, CA). Results from reads that could be uniquely mapped to a gene were used to calculate the expression level. FASTQC was used to check the quality of reads of all samples^1^. Raw data preprocessing was performed as previously described (Liu et al., 2019). The expression of each gene was normalized by the reads per kilobase per million mapped reads among different samples.

### Fluorescence Microscopy

For detection of subcellular localization by immunofluorescence, after fixation with 4% paraformaldehyde and permeabilization in 0.2% Triton X-100 (PBS), cells were incubated with the indicated antibodies for 8 h at 4°C, followed by incubation with TRITC-conjugated or FITC-conjugated secondary antibody for 1 h at 37°C. The nuclei were stained with DAPI. The images were visualized with a Zeiss LSM 510 Meta inverted confocal microscope.

### Real-Time Quantitative PCR Analyses

Total RNA was isolated using Trizol (Sigma, Saint Louis, MO) and cDNAs were synthesized by reverse transcription kit (Bio-Rad, Hercules, CA). Quantitative PCR reactions were performed using SYBR Green master mixture on HT7500 system (Applied Biosystems).

### Generation of Knock-Out Cells

The knock-out cell lines were generated using the Crispr-Cas9 method. Crispr guide sequences targeting *UBA3* was designed by software at http://crispr.mit.edu and clonedinto Lenti-Crispr pXPR_001. The sgUBA3 sequences was: 5′- CACCGTGAAGGGTCCAGATCGCTCG-3′. The MCF-7 cells were co-transfected with the Lenti-Crispr vector and packaging plasmids pVSVg and psPAX2. Puromycin-resistant single cells were plated in a 96-well dish to screen for positive monoclonal cells.

### Prediction of CpG Island and Methylation-Specific PCR

The CpG island of UBA3 gene was predicted by Database of CpG Islands^2^ and UCSC database^3^. Genomic DNA was extracted for methylation analysis from cells in culture by using Genomic DNA Miniprep Kit (sigma). One microgram of genomic DNA was modified with sodium bisulfite using the DNA Bisulfite Conversion Kit (TIANGEN) according to the specifications of the manufacturer. Methylation-specific PCR (MSP) was run in a total volume of 20 μl. MSP reactions were subjected to initial incubation at 95°C for 5 min, followed by 35 cycles of 94°C for 20 s, and annealing at the 60°C for 30 s and 72°C for 20 s. Final extension was done by incubation at 72°C for 5 min. MSP products were separated on 2% agarose gels and visualized after ethidium bromide staining. The following primers were used:

Unmethylated Forward 5′-TTAAAGTTTATGGGAGTTT AGTTGT-3′
Unmethylated Reverse 5′-CAAAATATATAAAAAATCCA AATCACTCA-3′
Methylated Forward 5′-TTAAAGTTTATGGGAGTTTA GTCGT-3′
Methylated Reverse 5′-ATATATAAAAAATCCAAATCGC TCG-3′

### The Human Protein Atlas Database

The Human Protein Atlas database^4^ was used to validate the protein expression level of UBA3 in breast cancer.

### The Cancer Genome Atlas (TCGA) Data

The mRNA data (RNA Seq v2), DNA methylation and clinical information for patients in TCGA-BRCA dataset were downloaded from https://www.synapse.org and cBioPortal database^5^, respectively and used for differential mRNA expression, correlation and gene set enrichment analysis.

### GEO Datasets Collection and Differential Expression Analysis

Microarray data were obtained from three datasets. The three series were accessed at the National Centers for Biotechnology Information (NCBI), Gene Expression Omnibus (GEO) database^6^ which served as a public repository for gene expression datasets, and the accession numbers were GSE66695 and GSE14088. Differentially expressed genes were obtained using GEO2R^7^. GEO2R is an interactive web tool that compares two groups of samples under the same experimental conditions and can analyze almost any GEO series.

### RNA Interference

Sequence information of the shRNAs are as follows:

sh*PTEN*: 5′-TGCAGATAATGACAAGGAA-3′;
sh*NC*: 5′-TTCTCCGAACGTGTCACGT-3′.

### Colony Formation Assay

Cells were seeded into 6-well plates with 600 cells per well. After 12 days, cells were fixed with 4% paraformaldehyde for 15min and stained with 0.5% crystal violet solution. The experiment was conducted in three independent triplicates.

### Cell Migration Assay

The assay was performed in an invasion chamber consisting of a 24-well tissue culture plate with 12 cell culture inserts (Becton–Dickinson). Briefly, cells (2 × 10^4^ per well) were seeded in the upper chambers in serum free cell culture medium (in triplicate), and medium containing 10% FBS was added to the bottom wells. Cells were allowed to migrate for 24–48 h in a humidified chamber at 37°C with 5% CO_2_. Then filter was removed and fixed with 4% formaldehyde for 20 min. Cells located in the lower filter were stained with 0.1% crystal violet for 15 min and photographed.

### Cell Proliferation Assay

Cell proliferation was assayed using the Cell Counting Kit-8 (CCK8) assay (Promega) according to the manufacturer’s protocol. The transfected cells were planted in 96-well plates (2,000 cells/well). Cell proliferation was detected every 24 h according to the manufacturer’s protocol. Briefly, 100 μl of 10% CCK8 solution was added to each well and incubated for 1 h at 37°C. The solution was then measured spectrophotometrically at 450 nm.

### Tumor Growth Assay

The experimental procedures in mice have been approved by the Animal Care and Use Committee of Academy of Military and Medical Sciences. BALB/c nude mice (6-weeks old, 18.0 ± 2.0 g) were obtained from Shanghai Laboratory Animal Center (SLAC, China). Cells (5 × 10^6^ per mouse) were inoculated subcutaneously into the right flank of the mice. Tumor size was measured every 2 days and converted to TV according to the following formula: TV (mm^3^) = (a × b^2^)/2, where a and b are the largest and smallest diameters, respectively. All animals were killed 4 weeks after injection, and the transplanted tumors were removed, weighed and fixed for further study.

### KEGG Pathway Enrichment Analysis

The online analysis tool DAVID (the Database for Annotation, Visualization and Integrated Discovery, Version 6.7) was used to determine the Kyoto Encyclopedia of Genes and Genomes (KEGG) (*P* < 0.05), in which we focused on the KEGG feature.

### Gene Set Enrichment Analysis

The association between phenotypes, pathways and UBA3/NEDP1 expression was analyzed using Gene Set Enrichment Analysis (GSEA v2.2)^8^. GSEA calculates a gene set Enrichment Score (ES) that estimates whether genes from pre-defined gene set [obtained from the Molecular Signatures Database (MSigDB)]^9^ are enriched among the highest- (or lowest-) ranked genes or distributed randomly. Default settings were used. Thresholds for significance were determined by permutation analysis (1,000 permutations). False Discovery Rate (FDR) was calculated. A gene set is considered significantly enriched when the FDR score is < 0.05.

### Statistical Analysis

Data were analyzed with GraphPad Prism 5 and SPSS 19.0 software. The statistical significance of differences between various groups was calculated with the Mann-Whitney or two-tailed, Student’s *t*-test and error bars represent standard deviation of the mean (SD). Data are shown as mean ± SD and *P* < 0.05 were considered to be statistically significant.

## Results

### MLN4924 Suppresses the Akt Signaling Pathway

**FIGURE 1:**
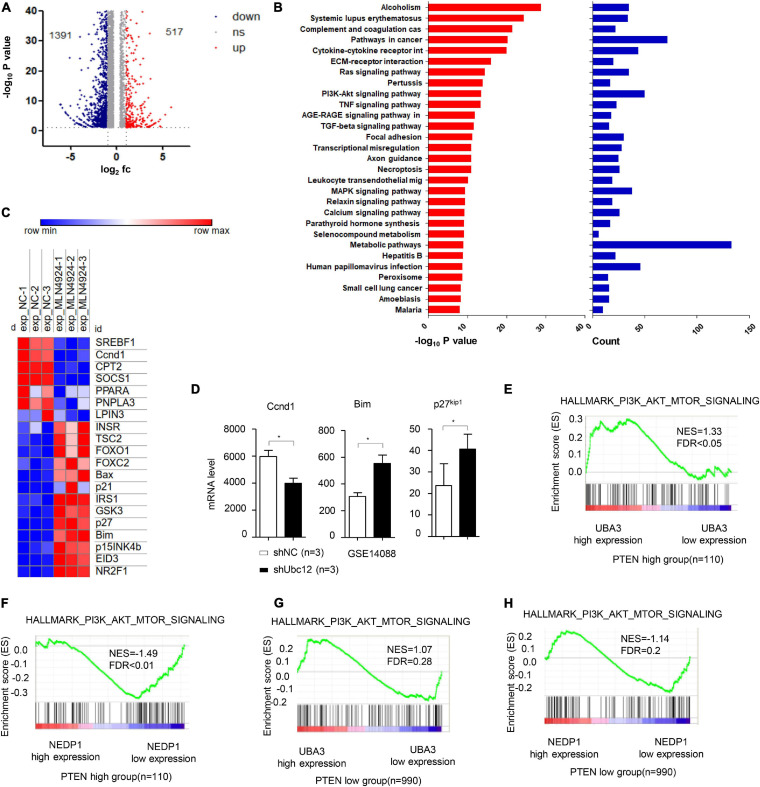
MLN4924 inhibits the PI3K/Akt signaling pathway. **(A)** Differentially expressed genes in MCF-7 cells between DMSO and MLN4924 (5 μM; 24 h) treatment groups are plotted as a volcano plot. **(B)** Bar charts depict the top ranked pathway analyzed from the KEGG pathway database. Blue (upper) and red (bottom) bars represent counts and significance (–log_10_ *P*-value), respectively. **(C)** Heat map of the downstream target genes of Akt signaling pathway between the DMSO and MLN4924 treatment group. **(D)** The mRNA level of Ccnd1, Bim and p27 were analyzed in Ubc12 knockdown NIH 3T3 cells. The GEO2R online tool was used to analyze differentially expressed genes on GSE14088 microarray. *P < 0.05, **P < 0.01, ***P < 0.001. **(E,F)** Enrichment plots of gene expression signatures for PI3K/Akt signaling according to UBA3 mRNA levels by gene set enrichment analysis (GSEA) of TCGA BRCA dataset in PTEN high expression group (NES = 1.33, FDR < 0.05) and PTEN low (with almost undetectable PTEN level) expression group (NES = 1.07, FDR = 0.28), respectively. Samples were divided into high and low UBA3 expression groups. False discovery rate (FDR) gives the estimated probability that a gene set with a given normalized ES (NES) represents a false-positive finding; FDR < 0.05 is a widely accepted cutoff for the identification of biologically significant gene sets. **(G,H)** Enrichment plots of gene expression signatures for PI3K/Akt signaling according to NEDP1 mRNA levels by gene set enrichment analysis (GSEA) of TCGA BRCA dataset in PTEN high expression group (NES = –1.49, FDR < 0.01) and PTEN low (with almost undetectable PTEN level) expression group (NES = –1.14, FDR = 0.2), respectively.

On the basis of high-throughput RNA-Seq, 1908 genes whose expression was changed in MCF-7 cells treated with MLN4924 were identified (Figure 1A and Supplementary Table 1). Notably, upon MLN4924 treatment, Kyoto Encyclopedia of Genes and Genomes (KEGG) pathway analysis indicated that the PI3K/Akt signaling pathway was one of the most significantly impacted pathways (Figure 1B). Furthermore, a heatmap analysis revealed that genes downstream of PI3K/Akt signaling, such as the cell cycle control protein cyclin D1 (Ccnd1), were robustly decreased upon MLN4924 treatment, while FOXO1, FOXO2, p21, Bax, Bim, p27^kip1^, p15^INK4b^ were increased (Figure 1C). Notably, a previous expression analysis showed that the PI3K/Akt pathway target gene Ccnd1 was decreased in NIH3T3 cells after knockdown of *Ubc12*, which encodes for a NEDD8-conjugating enzyme. Bim, a Bcl-2 family member, and p27^kip1^, one of the cyclin-CDK inhibitors, were both upregulated when Ubc12 was depleted (Figure 1D). These data indicate that MLN4924 inhibits the activity of the PI3K/Akt signaling pathway.

Next, we examined the potential correlation between PTEN expression levels and the effect of neddylation on the PI3K/Akt signaling pathway in breast cancer patients. Among breast cancer patients harboring high PTEN expression, the PI3K/Akt signaling pathway activation gene-set was markedly enriched in the genes encoding for the NEDD8-activating enzyme UBA3, whose expression was increased, and the deneddylase NEDP1, whose expression was decreased (Figures 1E,F). In contrast, among patients with low PTEN expression, there was no correlation between the PI3K/Akt signaling pathway activation gene set and UBA3 (Figure 1G) or NEDP1 (Figure 1H).

### PTEN Is Indispensable for MLN4924 Suppressing PI3K/Akt Signaling Pathway

**FIGURE 2:**
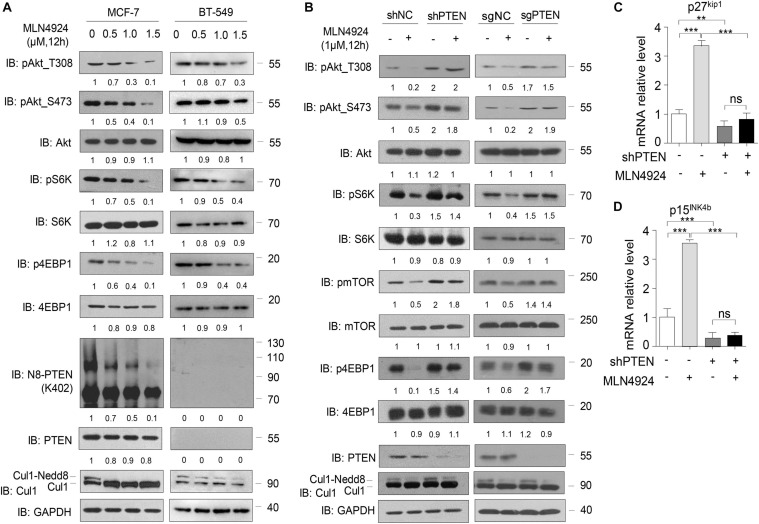
PTEN is indispensable for MLN4924 to inhibit PI3K/Akt signaling pathway activity. **(A)** Immunoblot of WCL from MCF-7 cells and BT-549 (*PTEN*-null) cells with MLN4924 treatment (1 μM) for 12 h. **(B)** Immunoblot of WCL from shNC/Control or shPTEN/sgPTEN MCF-7 cells. The cells were treated with 1 μM MLN4924 for 12 h. **(C,D)** Quantitative mRNA level analysis of p27^kip1^ and p15^INK4b^ in shNC and shPTEN MCF-7 cells. MLN4924 was used at indicated dose (1 μM; 12 h). The quantifications of indicated protein contents were analyzed. *P < 0.05, **P < 0.01, ***P < 0.001.

Consistent with our previous study (Xie et al., 2021), in MCF-7 cells, we observed a dose-dependent reduction in phosphorylation of Akt, S6K, 4EBP1, and mTOR within 12 h of exposure to MLN4924, while total protein levels of Akt, S6K, 4EBP1, and mTOR were unchanged (Figure 2A, left). PTEN neddylation on K402 was also decreased by MLN4924 treatment in a dose-dependent manner. However, in the PTEN-deficient breast cancer cell line BT-549, loss of PTEN negated the inhibitory effects of MLN4924 on PI3K/Akt signaling activity. In BT-549 cells, PTEN neddylation was undetectable under all conditions, and phosphorylation of Akt, S6K, 4EBP1, and mTOR was not decreased upon treatment with MLN4924 (Figure 2A, right).

Considering the facts that PTEN is a key upstream regulator of PI3K/Akt signaling, and that PTEN is covalently modified with Nedd8, we intended to further investigate whether MLN4924 regulates PI3K/Akt signaling via PTEN. To that end, we used a lentiviral strategy to establish an MCF-7 cell line with a stable knockdown of endogenous *PTEN* and the Lenti-crispr-cas9 method to create an MCF-7 line with a *PTEN* knockout. As shown in Figure 2B, silencing PTEN negated the inhibitory effects of MLN4924 on PI3K/Akt signaling activity. MLN4924 lost the ability to decrease the phosphorylation levels of Akt, S6K, 4EBP1, and mTOR in these lines. In addition, treatment of wild type cells with MLN4924 upregulated the Akt downstream genes p27^kip1^ and p15^INK4b^, and that effect was weakened when PTEN was depleted (Figures 2C,D). Hence, we conclude that PTEN is essential for inhibition of the activity of the PI3K/Akt signaling pathway by MLN4924.

### Involvement of PTEN in MLN4924-Mediated Inhibition of Breast Cancer Cell Growth and Migration

**FIGURE 3:**
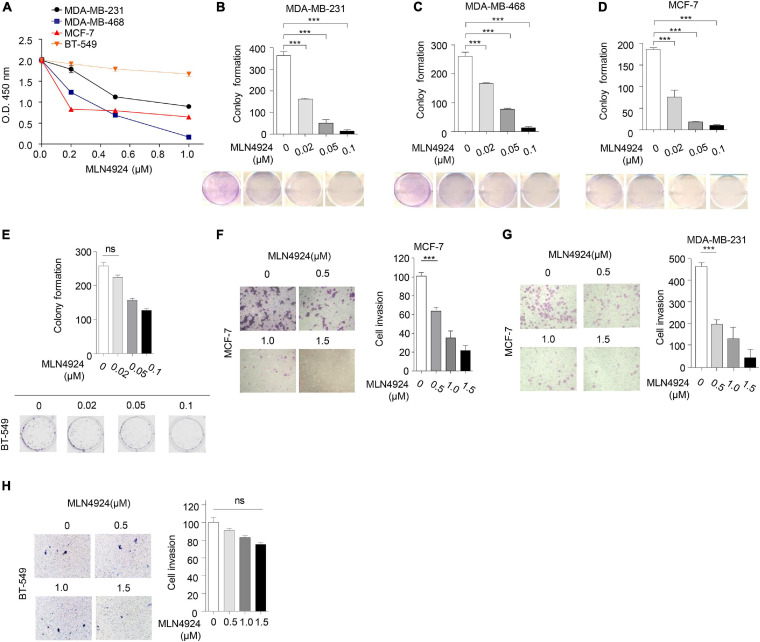
Anti-tumor effect of MLN4924 is dependent on PTEN. **(A)** Cells were treated with serial dilutions of MLN4924 for 72 h and cell viability was determined using CCK8 assays. Representative inhibitory curves from three independent experiments are shown for each cell line. **(B–E)** Cells were seeded into 6-well plates petri-dishes at 500 cells per dish in triplicate and treated with MLN4924 for 12 days, followed by 0.01% (w/v) crystal violet staining and colony counting. Representative images of three independent experiments are shown for colony formation. Image J was used to perform quantitative analysis. **(F–H)** Cells were treated with indicated concentrations of MLN4924 for 12 h before being subjected to trans-well migration analysis. Shown are representative images. Image J was used to perform quantitative analysis. Shown are representative images. Image J was used to perform quantitative analysis. Data are shown as the mean ± SD. *P*-values were calculated by one-way ANOVA test **(B–H)** and two-way ANOVA test **(A)**. *P < 0.05, **P < 0.01, ***P < 0.001.

**FIGURE 4:**
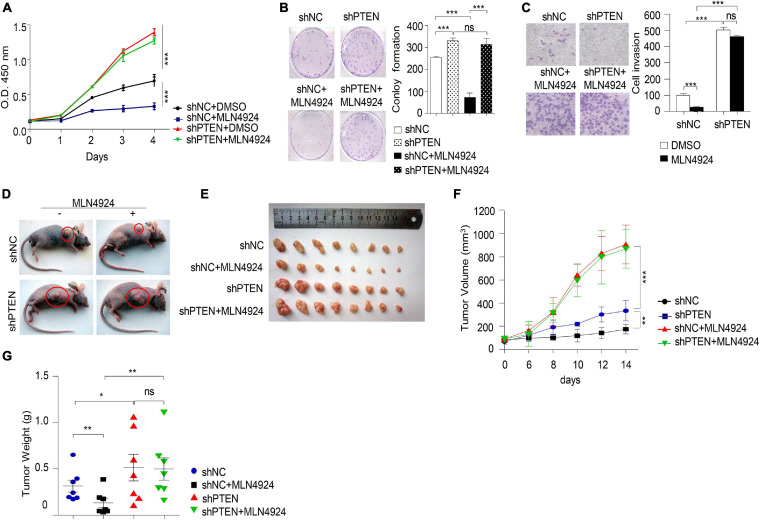
MLN4924 via PTEN to inhibit tumorigenesis. **(A–C)** Cell proliferation assay **(A)**, colony formation assay **(B)**, and cell migration assay **(C)** were analyzed in MCF-7 cells with the depletion of PTEN by shRNA. Cells were treated with 1 μM MLN4924 for 12 h before harvest. All data were representative of three independent experiments. Data shown are mean ± SD, *n* = 3 independent experiments. Image J was used to perform quantitative analysis. **(D–G)** Nude mice were injected subcutaneously for each of the indicated stable cell lines. Nude mice were treated with normal saline or MLN4924 (10 mg/kg i.p.) twice a day for 4 weeks. The transplanted tumors were removed and photographed **(D,E)**. Tumor volumes **(F)** and weights **(G)** were measured. Tumor weights and volumes are presented as mean ± SD; *P < 0.05, **P < 0.01, ***P < 0.001.

Next, we evaluated the ability of MLN4924 to reduce cell viability in different breast cancer cell lines. As shown in Figure 3A, MLN4924 showed a marked and dose-dependent reduction of cell viability in MDA-MB-231, MDA-MB-468, and MCF-7 cells. However, in BT-549 cells, which is a *PTEN* deficient cell line, MLN4924 did not indicate marked inhibition effect on cell proliferation. Moreover, MLN4924 clearly reduced clone formation in MDA-MB-231, MDA-MB-468, and MCF-7 cells (Figures 3B–D), except for in BT-549 cells (Figure 3E). Moreover, MLN4924 inhibited breast cancer cell migration, including MCF-7 cells and MDA-MB-231 cells (Figures 3F,G). In BT-549 cells, loss of *PTEN* correlated with an abrogation of the inhibitory effects of MLN4924 on tumor cell invasion (Figure 3H). Then, we generated stably transduced MCF-7 breast cancer cells by performing lentiviral transduction with Lenti-shNC (negative control), Lenti-shPTEN. It is worth noting that deletion of *PTEN* from MCF-7 cells abrogated the inhibitory effects of MLN4924 on cell proliferation (Figures 4A,B), and tumor invasion (Figure 4C). Moreover, we determined that loss of PTEN inhibited reduction of anchorage growth and tumor formation by MLN4924 in xenografts (Figures 4D–G). Collectively, we conclude that PTEN is indispensable for the tumor growth suppression activity of MLN4924.

### 2-DG Decreases PTEN Neddylation via Downregulating the mRNA Level of UBA3

Co-treatment with the glycolysis inhibitor 2-DG and MLN4924 greatly improves the efficacy of radiotherapy in breast cancer cells (Oladghaffari et al., 2017), suggesting an interplay between glucose metabolism and MLN4924-sensitive neddylation pathways. Importantly, breast cancer cells, like many other cancers, also exhibit an increased rate of glucose uptake (Martinez-Outschoorn et al., 2017). Our previous study showed that high concentrations of glucose trigger PTEN neddylation and that neddylated PTEN subsequently undergoes nuclear import (Xie et al., 2021). PTEN neddylation is known to promote the progression of breast cancer, thus providing another mechanistic link between glucose uptake and progression, but the mechanism by which glucose triggers PTEN neddylation remains unknown.

**FIGURE 5:**
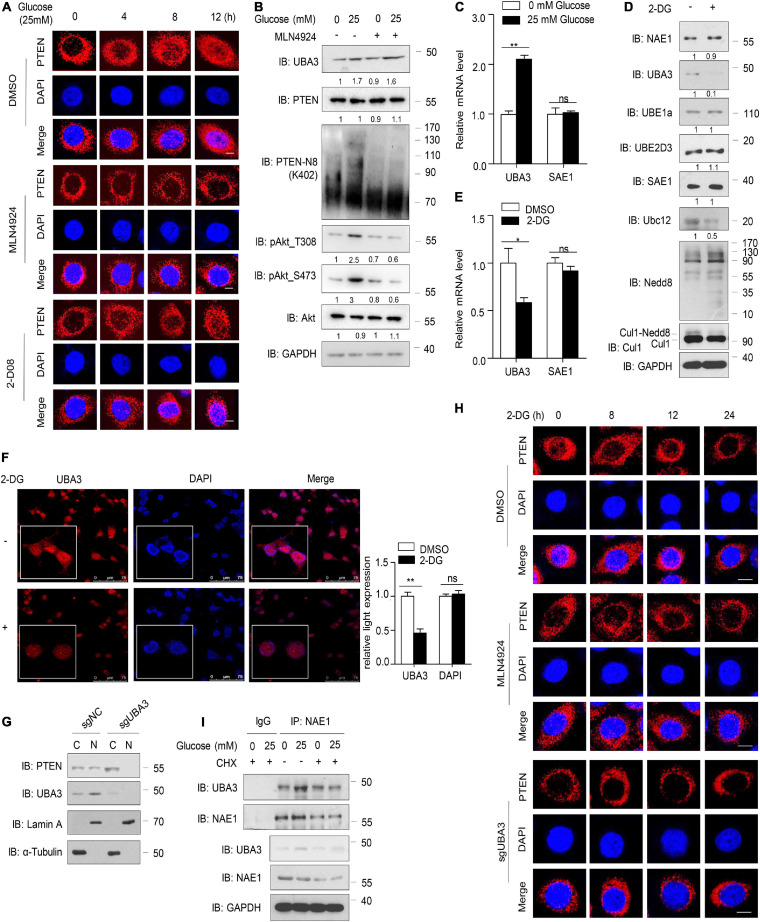
High concentration of glucose increases UBA3 expression. **(A)** Immunofluorescence of endogenous PTEN in MCF-7 cells. Cells were deprived for glucose for 12 h followed by glucose stimulation (25 mM) for different time. Cells were treated with DMSO, MLN4924 (1 μM; 12 h) or 2-D08 (5 mM; 24 h) before harvested, respectively. Scale bar, 25μm. **(B)** Immunoblot of WCL from MCF-7 cells cultured in cell medium containing different glucose concentrations. The cells were treated with DMSO or 1 μM MLN4924 for 12 h. The quantifications of indicated protein contents were analyzed. **(C)** UBA3 and SAE1 mRNA levels were analyzed by qPCR in MCF-7 cells cultured in different glucose concentrations. *P < 0.05, **P < 0.01, ***P < 0.001. **(D)** Immunoblot analysis of WCL from MCF-7 cells. MCF-7 cells were treated without or with 2-DG (5 mM, 24 h) before harvested. The quantifications of indicated protein contents were analyzed. **(E)** UBA3 and SAE1 mRNA levels were analyzed by qPCR in MCF-7 cells with or without 2-DG (5 mM; 72 h) treatment. Data are shown as the mean ± SD. *P < 0.05, **P < 0.01, ***P < 0.001. **(F)** Immunofluorescence of UBA3 in MCF-7 cells with or without 2-DG (5 mM, 24 h) treatment. Scale bar, 75 μm. The quantifications of indicated protein expression were analyzed. *P < 0.05, **P < 0.01, ***P < 0.001. **(G)** Cell fractionation assay of PTEN in UBA3 knockout MCF-7 stable cells. **(H)** Immunofluorescent assay of PTEN in MCF-7 or UBA3 knockout MCF-7 cells. Cells were deprived for glucose for 12 h followed by glucose stimulation (25 mM) for different time. MCF-7 cells were treated without or with 1 μM of MLN4924 for 12 h. Scale bar, 25μm. **(I)** Co-IP of interaction between UBA3 and NAE1 in MCF-7 cells. Anti-UBA3 immunoprecipitants were analyzed by western blotting with anti-NAE1 antibodies. Cells were deprived of glucose and followed by glucose stimulation (25 mM). Cells were treated with 100 ng/ml of CHX for 6 h.

In the present study, consistent with previous results, PTEN nuclear import increased in a time-dependent manner in the presence of high glucose concentrations (25 mM), but MLN4924 inhibited the accumulation of neddylated PTEN in the nucleus (Figure 5A). An inhibitor of SUMOylation, 2-D08 had no effect on glucose induced PTEN nuclear import (Figure 5A). Meanwhile, neddylated PTEN and the phosphorylation of Akt increased with the addition of glucose in the cell culture, but MLN4924 treatment abolished these effects (Figure 5B). Interestingly, we noticed that high glucose concentrations upregulated the protein level of UBA3 and this affect was unaffected by MLN4924 (Figure 5B). Then, our results showed that high glucose concentrations increased the mRNA level of UBA3, but not of the SUMO-activating enzyme SAE1 (Figure 5C). The glycolysis inhibitor, 2-DG, inhibited the expression of UBA3 and Ubc12, but not NAE1, Nedd8, SAE1, UBE1a or UBE2D3 (Figure 5D). The mono-neddylation of Cul1 was also declined under the treatment of 2-DG (Figure 5D). Importantly, we noticed that 2-DG treatment reduced the mRNA level of UBA3, not SAE1 (Figure 5E). Moreover, treatment with 2-DG did not affect the subcellular localization of UBA3, but the expression of UBA3 was reduced (Figure 5F). To investigate whether glucose regulates subcellular location of PTEN by increasing UBA3, we generated *UBA3* knock-out MCF-7 cells using the Crispr-Cas9 method. The result showed that PTEN was markedly retained in the cytoplasm in *UBA3*-deleted cells (Figure 5G). As shown in Figure 5H, 2-DG promotes PTEN nucleus export in a time dependent manner, and treatment with MLN4924 or the deletion of *UBA3* strengthened the accumulation of PTEN in the cytoplasm. Interestingly, the interaction of UBA3 and NAE1 was strengthened under the high glucose concentration (Figure 5I). We noticed that the formation of UBA3-NAE1 heterodimer was not strengthened under the treatment of CHX, an inhibitor of protein synthesis (Figure 5I). It seems like high glucose concentration triggered the mRNA expression of UBA3, and more UBA3 proteins were produced. Therefore, the enhanced interaction between UBA3-NAE1 is due to increased UBA3 expression rather than changes in protein conformation or activity. Hence, we suggest that glucose triggers PTEN neddylation and nuclear import by upregulating the expression of UBA3 and strengthened the interaction between UBA3-NAE1. However, we could not to rule out the possible change of Ubc12 to promote PTEN neddylation. The precise mechanism of how glucose enhances the expression of Ubc12 also needs further investigation.

### High Concentration of Glucose Inhibits UBA3 Promoter Methylation

**FIGURE 6:**
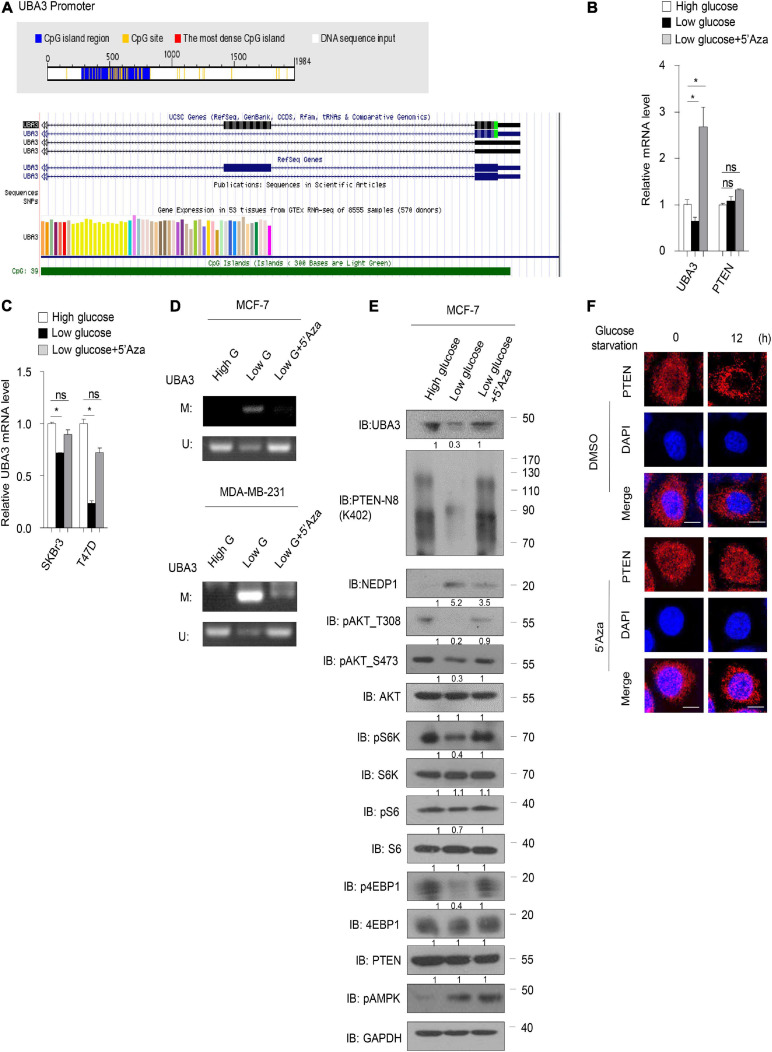
High concentration of glucose inhibits UBA3 promoter methylation. **(A)** The CpG Island within the UBA3 gene promoter region (upstream 2000bp of the UBA3). Blue line depicts the CpG island region and the yellow vertical bars represent a CpG site. White line depicts the DNA sequence (Top). The green line indicates the CpG Island of UBA3 in UCSC database (Bottom). **(B)** UBA3, NEDP1, PTEN expression was analyzed by qPCR in MCF-7 cells (high glucose, 25 μM; low glucose, 5 μM), without or with 5-Aza-2’-deoxycytidine (5’Aza) treatment. Cells were treated with 5 mM 5’Aza for 72 h, and the medium was replaced with freshly added 5’Aza for every 24 h. Data are shown as the mean ± SD. *P < 0.05, **P < 0.01, ***P < 0.001. **(C)** UBA3 mRNA level was analyzed by qPCR in breast cancer cell lines. Data are shown as the mean ± SD. *P < 0.05, **P < 0.01, ***P < 0.001. **(D)** The methylation status of the UBA3 promoter in breast cancer cell lines medium containing different glucose concentrations, without or with 5’Aza treatment. The CpG islands of UBA3 were analyzed by methylation-specific PCR (MSP). U, unmethylated; M, methylated. **(E)** Immunoblot analysis of WCL from MCF-7 cells cultured in cell medium containing different glucose concentrations, without or with 5’Aza treatment. The quantifications of indicated protein contents were analyzed. **(F)** Immunofluorescence of PTEN in MCF-7 cells. Cells were deprived for glucose for different time. Cells were treated without or with 1 μM 5’Aza for 12 h. Scale bar, 25 μm.

Next, we sought to determine the underlying mechanism by which glucose regulates the UBA3 mRNA level. Sequence analyses using the Database of CpG Islands (see text footnote 2) and the University of California, Santa Cruz Genome Browser database (see text footnote 3) revealed that a CpG island is located within the UBA3 promotor region (Figure 6A). Low glucose reduced the mRNA level of UBA3 in breast cancer cells (Figure 6B). However, treatment with 5-azacytidine (5’Aza), a DNA methylation inhibitor, reversed that effect. As a comparison, PTEN was not affected by lowing the glucose concentration or by the 5’Aza treatment. Similar results were found in other breast cancer cells (Figure 6C). Then we performed a methylation-specific PCR assay to clarify whether glucose regulates the UBA3 promoter methylation in breast cancer cells. The data indicated that UBA3 promoter methylation was increased under low glucose concentrations and that 5’Aza treatment reversed this trend (Figure 6D). Moreover, lowering glucose concentrations correlated with lower UBA3 protein levels, PTEN neddylation levels and the Akt/mTOR signaling pathway activity (Figure 6E). Glucose starvation-induced PTEN nuclear export was abolished by 5’Aza treatment (Figure 6F). Taken together, our data suggest that high glucose concentrations might increase UBA3 mRNA and promote PTEN neddylation via inhibition of methylation of the UBA3 promoter.

### UBA3 Expression Is Negative Correlated With Hypomethylation of Upstream CpG-Island in Breast Cancer

**FIGURE 7:**
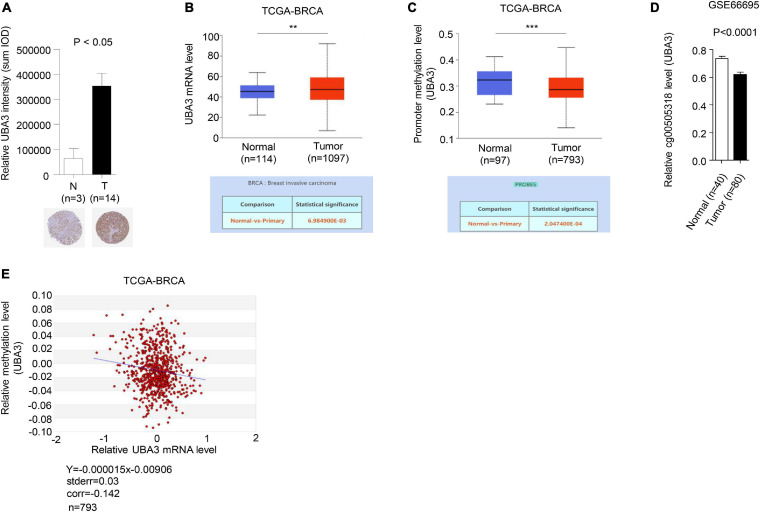
UBA3 is upregulated and negative correlated with its promoter methylation level in breast cancer. **(A)** Representative images from immunohistochemical staining of UBA3 in breast tumors and matched adjacent tissues. The relative UBA3 expressions are shown as histogram. *P*-values were calculated by Student’s *t*-test. *P < 0.05. Data are shown as the mean ± SD. **(B)** The mRNA levels of UBA3 between normal and tumor tissues in the TCGA cohort of BRCA samples. *P < 0.05, **P < 0.01, ***P < 0.001. **(C)** The methylation levels of UBA3 between normal and tumor tissues in the TCGA cohort of BRCA samples. *P < 0.05, **P < 0.01, ***P < 0.001. **(D)** The relative promoter methylation levels of UBA3 between normal and breast cancer tissues in the GEO database (GSE66695). **(E)** The correlation between methylation percentage and mRNA levels of UBA3 in the TCGA cohort of BRCA samples. The blue line shows the linear regression of methylation percentage and mRNA expression (Log2).

To further investigate the relevance of the connections between promoter methylation and transcriptional activity of UBA3 in breast cancer, the expression and promoter methylation levels of UBA3 were analyzed in the Human Protein Atlas, the database of the Cancer Genome Atlas Program of the National Cancer Institute (CGAP), and the Gene Expression Omnibus (GEO) database. The expression of UBA3 was found to be upregulated in tumor tissues relative to adjacent normal tissues (Figures 7A,B). Conversely, promoter methylation of UBA3 is downregulated in breast cancer (Figures 7C,D). A correlation assay between methylation and mRNA level of UBA3 was also performed utilizing the MethHC database. A linear regression demonstrated that UBA3 mRNA levels are negatively correlated with promoter methylation levels (Figure 7E). These data demonstrate that promoter hypomethylation results in UBA3 overexpression in breast cancer.

## Discussion

Neddylation is necessary for development, and overactivation of neddylation often leads to tumorigenesis (Huang et al., 2009; Zhou et al., 2018, 2019). MLN4924 is an effective small molecule inhibitor of NEDD8-activating enzyme E1, and this molecule has been evaluated in a series of phase 1, 2, and 3 clinical trials for oncotherapy (Soucy et al., 2009; Zhou and Jia, 2020). Recently, PTEN, a well-known tumor suppressor, was identified as a novel target for modification with NEDD8, and PTEN neddylation has been associated with tumor development in all types of breast cancers (Xie et al., 2021). PTEN loss of function is one of the most common events observed in multiple cancers (Hollander et al., 2011), and we previously reported that neddylation is a key regulatory mechanism that leads to loss of the tumor-suppressive function of PTEN and activation of Akt signaling pathways. Meanwhile, PTEN expression is often associated with anti-tumor drug resistance (Wein and Loi, 2017). Thus, we hypothesized that PTEN status might affect the anti-tumor effectiveness of MLN4924.

In this study, we found that MLN4924 strongly inhibits the PI3K/Akt signaling pathway in breast cancer. The neddylation pathway is positively correlated with Akt signaling pathway activity in patients with high PTEN expression but not in low expression patients according to the Cancer Genome Atlas Breast Invasive Carcinoma dataset. Moreover, PTEN loss abolished the anti-tumor and Akt signaling inhibitory effects of MLN4924. These data imply that PTEN neddylation may be the crucial therapeutic target of MLN4924 in breast cancer. Taken together, PTEN loss may act as a driver of MLN4924 resistance in breast cancer, and this study may thus provide a more focused treatment strategy.

Neddylation affects numerous important biological processes, such as cell cycle progression (Jia et al., 2011; Luo et al., 2012; Mackintosh et al., 2013; Yao et al., 2014; Chen et al., 2016). Cullins have been reported as major substrates for neddylation, but a growing number of non-Cullin targets of Nedd8 have also been identified, including p53, Smurf1 and PTEN (Xirodimas et al., 2004; Xie et al., 2014, 2021). Although the promotion of tumor growth by neddylation has been established, mechanisms by which neddylation promotes tumorigenesis are still poorly understood. More importantly, the physiological conditions leading to neddylation pathway activation remain unclear.

Recently, we reported that high glucose triggers UBA3 upregulation and downstream PTEN neddylation in breast cancer (Xie et al., 2021). In the present report, we found that high glucose increased UBA3 mRNA levels by inhibiting UBA3 promoter methylation in breast cancer cells. High glucose concentration triggered the expression of UBA3 and enhanced the formation of heterodimer with NAE1. Once the cells were treated with CHX, the interaction between UBA3-NAE1 could not increase even under the high glucose concentration, which indicated that the strengthened UBA3-NAE1 heterodimer was due to increased UBA3 expression rather than changes in protein conformation or activity. Moreover, the mRNA level of UBA3 is negatively correlated with UBA3 promoter methylation level in breast cancer patients. A previous study showed that high glucose triggers cytoplasmic translocation of a key DNA methylase, DNMT3A, and reduces the level of DNA promoter methylation (Zhang et al., 2016). DNMT3A is essential for genome regulation and development and has been associated with tumorigenesis, and structural studies have indicated an enzymatic preference of DNMT3A for CpG sites of target genes (Zhang et al., 2018). Therefore, we conjecture that glucose may upregulate via promoter hypomethylation UBA3 induced by cytoplasmic translocation of DNMT3A; this proposed mechanism calls for additional study.

## Conclusion

In conclusion, these results in this work support a mechanism in which high concentrations of glucose activate a neddylation pathway via downregulation of UBA3 promoter methylation, and this mechanism may be closely correlate with the overactivation of the neddylation pathway in cancers. In addition, to our knowledge, this is the first report on physiological conditions leading to neddylation activation. We also demonstrate that targeting the neddylation pathway is an attractive therapeutic approach for breast cancer patients with PTEN expression. Our findings provide insight into the clinical significance of MLN4924 precision therapies in breast cancer, and PTEN neddylation may be a useful marker to guide MLN4924 therapy for breast cancer in the future.

## Data Availability Statement

The original contributions presented in the study are publicly available. This data can be found here: https://www.ncbi.nlm.nih.gov/sra/PRJNA723112; accession number: PRJNA723112.

## Ethics Statement

The animal study was reviewed and approved by the Institutional Animal Care and Use Committee (IACUC) at the Beijing Institute of Lifeomics.

## Author Contributions

PX, MD, ZP, WG, FL, WL, YC, HL, XZ, and CL performed the experiments. PX and HJ designed the experiments and analyzed the data. PX, ZP, WG, and MD co-wrote the manuscript. PX, LZ, and HJ conceived the idea and supervised the study. All authors contributed to the article and approved the submitted version.

## Conflict of Interest

The authors declare that the research was conducted in the absence of any commercial or financial relationships that could be construed as a potential conflict of interest.

## Footnotes

1 http://www.bioinformatics.babraham.ac.uk/projects/fastqc

2 http://dbcat.cgm.ntu.edu.tw/

3 http://genome.ucsc.edu/index.html

4 https://www.proteinatlas.org/

5 www.cbioportal.org

6 http://www.ncbi.nlm.nih.gov/geo/

7 http://www.ncbi.nlm.nih.gov/geo/geo2r/

8 http://www.broad.mit.edu/gsea/

9 http://software.broadinstitute.org
